# Electrophysiology and cardiac device specialist exams: Why allied health professionals should aim for certification and how to get it

**DOI:** 10.1016/j.hrcr.2023.08.002

**Published:** 2023-09-15

**Authors:** Stacey Wendling

**Affiliations:** Covenant Healthcare, Saginaw, Michigan

**Keywords:** Allied health professional, Exam, Certification, CEPS, CCDS, CDRMS

“The mission of the International Board of Heart Rhythm Examiners (IBHRE) is to promote optimal outcomes for heart rhythm patients.”^1^ Specialty-focused exams are now offered to allied health professionals (nurse, nurse practitioner, physician assistant, lab tech, etc) and award certification to those that successfully pass the exam while validating individuals as unparalleled experts in heart rhythm management.

### Who should apply

The focus population for the creation of the electrophysiology (EP) specialist exams is vast, covering many specialties including nursing, EP lab/device technicians, industry representatives, and education/research as long as individuals partake in the care of patients with cardiac arrhythmias.

### Why become certified

“Master this unique subspecialty”^1^ by earning distinguished international recognition for advanced education and clinical skills in the care of patients with cardiac arrhythmias. Though certification is not required to work in a cardiac care setting, the board highlights that specialty certification “is an investment in one’s career and one’s professional and personal development.”^1^

The 2023 HRS/EHRA/APHRS/LAHRS expert opinion statement on the practical management of remote monitoring clinics^2^ recommends that those interpreting, reporting, and billing for remote monitoring should be certified, including with the option of IBHRE. It further recommends that the institution or practice that the clinician is working for should support the maintenance of that certification.

### How to get certified

“The exams test competencies necessary to provide quality patient care in cardiac pacing, electrophysiology, and device remote monitoring.”^1^ Certification options and eligibility requirements for allied professionals are summarized in Table 1.

**Table 1:** Certification options and eligibility requirements for allied professionals

CCDS	CEPS	CDRMS
Formal training program in cardiac pacing and/or EP *and* minimum of 6 months didactic training + 6 months exposure to the field	Minimum of 2 years of clinical experience in the field of EP	Exposure to all major manufacturer’s devices is highly encouraged Minimum of an associate degree in a medical field + 1 year of CIED remote monitoring
OR: Pass the RCES or RCIS exams *and* a minimum of 12 months of clinical experience	OR: Formal comprehensive training program in cardiac pacing and/or electrophysiology *and* 6+ months clinical experience	OR: Minimum of 2 years of experience with CIED remote monitoring or in-person evaluation
OR: Two years of experience or 100 cases in cardiac pacing and/or EP with direct exposure to patients	OR: Pass the RCES exam *and* 12 months clinical EP experience	OR: Pass the RCES or RCIS exam *and* at least 1 year of CIED remote monitoring or in-person evaluation
Provide a letter of recommendation attesting to the candidate’s current or past involvement in the field	Provide a letter of recommendation attesting to the candidate’s current or past involvement in the field	Provide a letter of recommendation that validates your commitment in the field

CCDS = Certified Cardiac Device Specialist; CDRMS = Cardiac Device Remote Monitoring Specialist; CEPS = Certified Electrophysiology Specialist; CIED = cardiac implantable electronic device; EP = electrophysiology; RCES = Registered Cardiac Electrophysiology Specialist; RCIS = Registered Cardiovascular Invasive Specialist.

Exams are written and developed by writing committees composed of IBHRE members and subject matter experts in cardiology and are completed at a local Prometric test center. An exam fee discount is available to Heart Rhythm Society members.

Exam topics/questions strive to draw out evidence of the high level of competency expected of an arrhythmia specialist by drawing attention to areas of fundamentals of electrophysiology and electronics (devices) as well as the immensely multifaceted topic of rhythm recognition.

Though not explicitly complete, the following list, as taken from the IBHRE handbook,^1^ outlines the main focus areas of content for the Certified Cardiac Device Specialist (CCDS), Cardiac Device Remote Monitoring Specialist (CDRMS), and Certified Electrophysiology Specialist (CEPS) allied health certification exams:

• Physics of electrophysiology
• Cardiac anatomy and physiology
• Pharmacology
• Fundamentals of electrophysiology
• Electrocardiogram and recognition of dysrhythmias
• Clinical assessment, laboratory considerations (CEPS)
• Invasive electrophysiology (CEPS)
• Implantable devices
• Applied science and technology (CCDS)
• Device and lead function/malfunction (CDRMS)
• Remote service management, device monitoring (CCDS, CDRMS)
• Real-time and diagnostic imaging
• Research methodology and interpretation

Test candidates are encouraged to use exam prep resources provided on the IBHRE website, including suggested reading lists and links to websites providing further educational content. The importance of resourcing various textbooks, cardiac journals and articles, and individual clinical cardiac experience is emphasized. Consulting with a mentor from the IBHRE Ambassador program provides anecdotal advice derived from the personal experience of a highly knowledgeable and certified heart rhythm practitioner.

### How to maintain certification

The IBHRE has transitioned to a new approach of longitudinal competency validation through the C3 process: Continuous Competency, Continuous Learning, and Continuous Certification. The completely virtual process replaces the 10-year comprehensive exam requirement with a series of 12 brief online assessments to be completed every 2 years to maintain certification.

### Should you take the exam?

Whether you pursue certification to gain a competitive career advantage, to elevate professional status, or purely to increase personal knowledge, the result of obtaining specialty certification for allied health professionals aligns with the mission of the IBHRE: to facilitate optimal outcomes for heart rhythm patients. If you are in the profession of caring for cardiac arrhythmia patients, certification is for you.
